# LEP-AD: language embedding of proteins and attention to drugs predicts drug-target interactions

**DOI:** 10.1186/s13321-026-01167-9

**Published:** 2026-04-27

**Authors:** Reem Alsulami, Robert Lehmann, Anuj Daga, Sumeer A. Khan, Raik Grünberg, Ahmed Abogosh, David Gomez Cabrero, Stefan T. Arold, Robert Hoehndorf, Jesper Tegner, Narsis A. Kiani

**Affiliations:** 1 Computer, Electrical and Mathematical Sciences and Engineering Division, King Abdullah University of Science and Technology (KAUST), 23955-6900 Thuwal, Saudi Arabia; 2 Unit of Computational Medicine, Department of Medicine, Center for Molecular Medicine, Karolinska Institutet, Karolinska University Hospital, L8:05, SE-171 76 Stockholm, Sweden; 3 Division of Biomedical Sciences, King Abdullah University of Science and Technology (KAUST), 23955-6900 Thuwal, Saudi Arabia; 4 Science for Life Laboratory, Tomtebodavagen 23A, 17165 Solna, Sweden; 5 Translational Bioinformatics Unit, Navarrabiomed, Universidad Pública de Navarra (UPNA), IdiSNA, Pamplona, Spain; 6 Algorithmic Dynamic Lab, Center for Molecular Medicine, Karolinska Institute, Stockholm, Sweden; 7 Department of Oncology-Pathology, Karolinska Institutet, Stockholm, Sweden; 8 KAUST Center of Excellence for Smart Health, King Abdullah University of Science and Technology (KAUST), 23955-6900 Thuwal, Saudi Arabia

**Keywords:** Drug target affinity, DTI, Drug discovery, Binding affinity, ESM, GAT, GCN

## Abstract

**Introduction:**

Predicting drug–target interactions remains a significant challenge in drug development and lead optimization. Recent advances have leveraged machine learning algorithms to model drug–target interactions from molecular and sequence data.

**Materials and Methods:**

In this work, we use Evolutionary Scale Modeling (ESM-3) to construct a transformer-based protein language representation for drug–target interaction prediction. We introduce LEP-AD (Language Embedding of Proteins and Attention to Drugs), a modular architecture that combines pretrained protein language models with graph-based molecular encoders to predict binding affinity values.

**Results:**

We systematically benchmark LEP-AD alongside a range of established deep learning methods across multiple datasets—Davis, KIBA, DTC, Metz, ToxCast, and STITCH. To assess predictive validity, we compare model-derived rankings of drug–target interactions with experimental results reported in the literature. In addition, we perform new experimental assays to evaluate the binding of three ATP-competitive Src kinase inhibitors—Dasatinib, UM-164, and Saracatinib—where experimentally measured IC₅₀ and pKᵢ values are consistent with the predicted rankings.

**Conclusion:**

In summary, our benchmark highlights the strengths and limitations of current drug–target interaction models across diverse datasets and evaluation settings. The results emphasize the impact of pretrained protein and molecular representations on predictive performance and illustrate the persistent challenges of generalization, while the modular LEP-AD framework provides a flexible reference point for comparative evaluation.

**Scientific Contribution:**

This study presents LEP-AD, a modular deep learning framework for drug–target interaction prediction that integrates pretrained protein language representations with graph-based molecular encoders. Beyond introducing the architecture, we provide a systematic benchmark under similarity-aware evaluation settings and experimental validation, highlighting the impact of pretrained protein embeddings on predictive behavior across diverse datasets.

**Supplementary Information:**

The online version contains supplementary material available at 10.1186/s13321-026-01167-9.

## Introduction

Successful drug development necessitates a deep understanding of the molecular mechanisms underlying a drug's action, especially regarding Drug-Target Interactions (DTIs). Identifying a drug's binding affinity and selectivity to target proteins is pivotal in gauging efficacy and predicting potential side effects from off-target interactions. Thus, reliable prediction of DTIs is central to reducing the cost, time, and failure rate of drug development.

Addressing the challenges posed by the heterogeneous nature of targets, similarity between targets and off-targets, data sparsity, inter-individual variability, and incomplete molecular understanding, recent advances have emerged from computational methods integrating molecular modeling, simulations, and experimental data. While molecular dynamics and quantum mechanics can provide reliable results [1, 2], their high computational cost limits their use in high-throughput screening. Conversely, methods based on molecular docking are less demanding computationally but suffer from low accuracy. An increasing number of investigators have explored deep learning methods as they provide a trade-off between computational cost and accuracy [3–5]. Early approaches primarily relied on convolutional neural networks (CNNs) applied to one-dimensional protein sequences and string-based drug representations such as SMILES [3, 6]; although effective at capturing local motifs and substructures, these representations do not preserve the topological or three-dimensional information necessary for accurately modeling molecular interactions. Large-scale transformer models trained on massive protein sequence corpora and chemical libraries have enabled contextual, high-capacity embeddings that encode evolutionary, structural, and functional information, providing richer features for downstream DTI tasks [4, 7, 8]. In parallel, the emergence of graph neural networks (GNNs) has addressed long-standing limitations of earlier models by representing small molecules as graphs, where atoms and bonds are treated as nodes and edges. Variants such as graph convolutional networks (GCNs), graph isomorphism networks (GINs), and especially graph attention networks (GATs) learn chemically meaningful representations directly from molecular topology, overcoming the constraints of manually crafted descriptors [9, 10]. Although predicted protein structures from AlphaFold have expanded opportunities for structure-aware modeling, these models often lack bound ligands and may not capture the conformations most relevant for binding. Consequently, attention-based graph architectures and cross-attention mechanisms between drug and protein representations have gained traction as flexible, end-to-end approaches capable of highlighting putative binding-relevant substructures and residues [5]. Collectively, these developments reflect a broader movement away from hand-engineered features toward more comprehensive and biologically grounded representation learning in DTI prediction.

Despite recent progress using deep learning-based methods, challenges remain. Existing approaches vary widely in how they represent proteins and small molecules, how they model interactions, and how they are evaluated. Although many models achieve high performance under random splits, their generalization ability has not been widely investigated in settings where the test drugs and proteins differ substantially from those seen during training [3]. This gap raises important questions about the true robustness of current DTI predictors and about the relative contributions of representation quality, architectural complexity, and data similarity to overall model performance. To address these gaps, we introduce LEP-AD, a model that leverages ESM-3 protein embeddings, which encode rich sequence, structural, and functional information learned from large-scale self-supervised training [7]. By integrating these biologically grounded protein representations with graph-based drug encodings, LEP-AD allows us to systematically examine the extent to which pretrained molecular and protein features contribute to DTI prediction accuracy under similarity-aware evaluation settings.

We evaluate LEP-AD against a range of recently proposed deep learning models for drug–target interaction prediction, which differ primarily in their strategies for encoding molecular structures and protein sequences and for integrating these representations. All evaluations are conducted under similarity-aware data splits that enforce meaningful separation between training and test drugs and proteins. Transformer-based approaches such as DTIAM [4] use large-scale self-supervised pretraining to generate contextual embeddings for both proteins and drug substructures, while models like TDGraphDTA [11] and CSDTI [12] combine graph neural networks for molecular representation with convolutional encoders for proteins, using cross-attention to capture fine-grained interaction patterns. Other architectures, including GTB_DTI [3] and IMAEN [13], employ multi-scale CNNs or self-attention–enhanced encoders for proteins together with graph-based or sequence-based molecular embeddings, fusing these representations through multilayer perceptrons for downstream affinity prediction.

While existing approaches have significantly advanced our ability to predict drug–target interactions, they often remain purely predictive tools without accompanying experimental validation. Without experimental confirmation, the reliability and translational potential of these predictions remain uncertain. For example, two methods may produce similar overall statistical scores, yet their ranking of candidate targets can differ substantially. This observation underscores the importance of independent experimental validation. Such validation ensures that computational predictions translate into actionable insights in an industrial drug discovery setting, providing the empirical evidence needed to prioritize drug candidates, refine models, and ultimately bridge the gap between computational innovation and clinical application. Therefore, we conduct new biological experiments validating the predicted binding—across all evaluated deep learning methods, including LEP-AD—of three ATP-competitive Src kinase inhibitors.

Our findings indicate that no particular architectural modification reliably delivers superior performance across all tasks. Rather, predictive accuracy varies considerably across datasets and data splits, highlighting the dataset-dependent nature of DTI modeling. All methods exhibit a noticeable drop in performance under the dissimilar split, where proteins and drugs are unseen during training, indicating a generalizability issue that is likely attributable to data limitations.

## Results

### LEP-AD accurately predicts drug-target interaction affinities

**Fig. 1:**
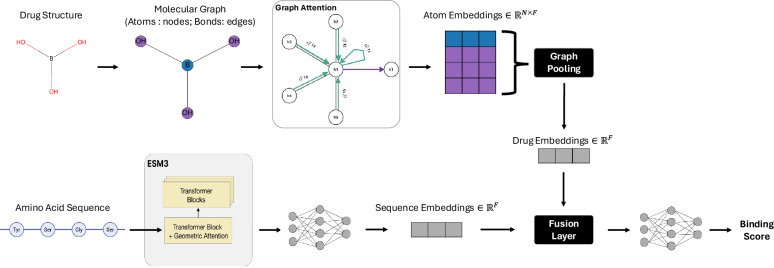
LEP-AD architecture for drug–protein binding affinity prediction. The drug is encoded as a molecular graph and processed by a graph attention network to obtain atom-level embeddings, which are aggregated via graph pooling to form a drug representation for concatenation- or summation-based fusion. In parallel, the protein sequence is encoded using a pretrained ESM-3 model to produce a sequence-level embedding. Drug and protein representations are integrated through a fusion layer and passed to a multilayer perceptron to predict the binding score. For cross-attention-based fusion, the full atom-level graph representation is used directly

This study evaluates a representation-learning framework for predicting protein–ligand binding affinity (Fig. 1). The model integrates two complementary modules that generate feature representations for drug and protein inputs (see Methods). Each drug or small molecule is initially represented using SMILES notation, a text-based format that encodes its structural topology. These SMILES strings are converted into molecular graphs using RDKit [14], where atoms correspond to nodes and chemical bonds to edges. The molecular graph is then processed using a graph attention–based neural network [15, 16], which models the topological and chemical context of each compound by applying attention mechanisms over atom–bond interactions. For proteins, we leverage ESM-3 [7], a large-scale protein language model that captures rich sequence, structural, and functional information learned through self-supervised training, enabling biologically grounded protein representations.

The performance of LEP-AD is evaluated across six benchmark datasets: Davis [17], KIBA [18], Drug Target Commons (DTC) [19], Metz [20], ToxCast [21], and STITCH [22]. Model effectiveness is assessed using three complementary metrics—the Concordance Index (CI), ${r}_{m}^{2}$, and Mean Squared Error (MSE)—which together quantify ranking capability, explained variance, and prediction error (see Methods). For comparative analysis, we benchmark LEP-AD against a set of contemporary deep learning models published between 2023 and 2025, together with two conventional machine-learning algorithms—Random Forest and XGBoost regressors—that are commonly used for binding affinity prediction. Four deep learning baselines—TDGraphDTA [11]**,** CSDTI [12], IMAEN [13], and GTB-DTI [3]—are drawn from a recent comprehensive benchmark study that evaluated more than 20 architectures spanning convolutional, transformer-based, and graph neural network approaches. We additionally include DTIAM [4], a unified transformer-based framework for drug–target interaction and mechanism-of-action prediction. Collectively, these baselines capture the dominant modeling paradigms currently employed in drug–target affinity prediction. Additional methodological details for each baseline are provided in the Methods section.

**Fig. 2:**
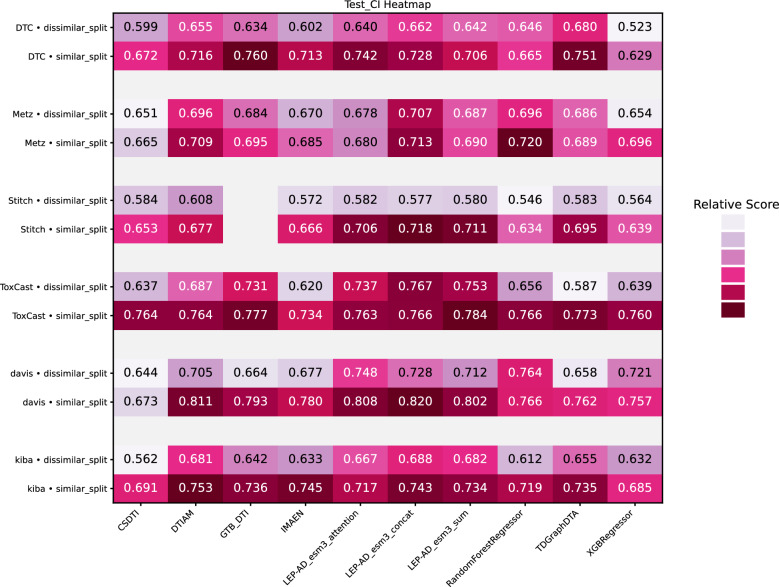
Benchmark comparison using the concordance index (CI) as a metric across all datasets and cross-validation splits. The heatmap shows the CI performance of all evaluated models—including CSDTI, DTIAM, GTB_DTI, IMAEN, LEP-AD (three fusion variants), Random Forest Regressor, TDGraphDTA, and XGBoost Regressor—across both similar and dissimilar splits for each dataset (DTC, Metz, Stitch, ToxCast, Davis, and KIBA). Colors represent per-dataset normalized scores, enabling performance comparison across models within each dataset. Original CI values are annotated inside the cells, and darker cells indicate better performance

**Fig. 3:**
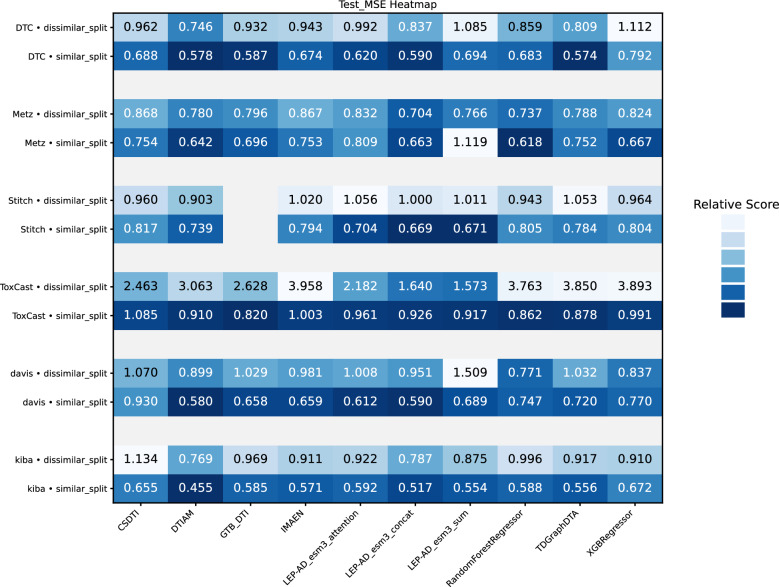
Benchmark comparison of mean squared error (MSE) across all datasets and cross-validation splits. The heatmap shows the MSE performance of all evaluated models—including CSDTI, DTIAM, GTB_DTI, IMAEN, LEP-AD (three fusion variants), Random Forest Regressor, TDGraphDTA, and XGBoost Regressor—across both similar and dissimilar splits for each dataset (DTC, Metz, Stitch, ToxCast, Davis, and KIBA). Colors represent per-dataset normalized scores, enabling performance comparison across models within each dataset. Original MSE values are annotated inside the cells, and darker cells indicate better performance

**Fig. 4:**
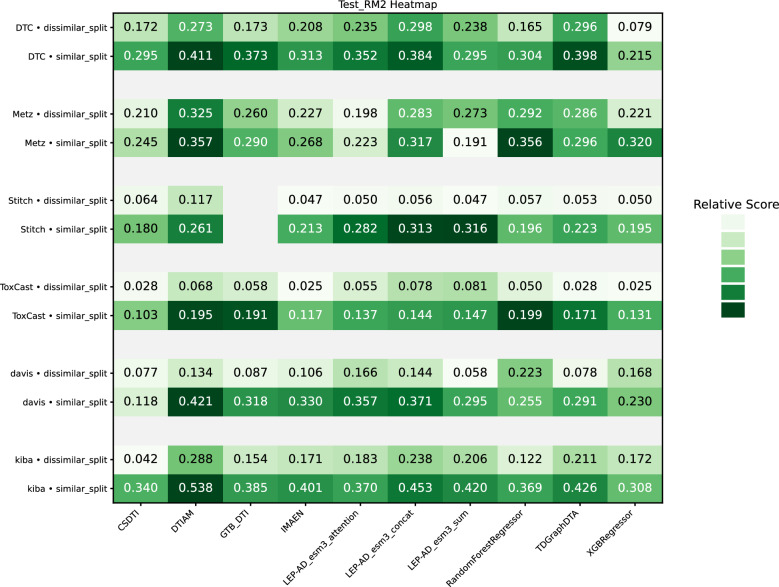
Benchmark comparison of ${\mathrm{r}}_{\mathrm{m}}^{2}$ across all datasets and cross-validation splits. The heatmap shows the ${\mathrm{r}}_{\mathrm{m}}^{2}$ performance of all evaluated models—including CSDTI, DTIAM, GTB_DTI, IMAEN, LEP-AD (three fusion variants), Random Forest Regressor, TDGraphDTA, and XGBoost Regressor—across both similar and dissimilar splits for each dataset (DTC, Metz, Stitch, ToxCast, Davis, and KIBA). Colors represent per-dataset normalized scores, enabling performance comparison across models within each dataset. Original ${\mathrm{r}}_{\mathrm{m}}^{2}$ values are annotated inside the cells, and darker cells indicate better performance

Across all three benchmark metrics (Figs. 2, 3 and 4), we observe a consistent pattern: All models achieve substantially higher performance under the similar split, where training and test sets include chemically and evolutionarily related compounds and proteins. Performance drops markedly under the dissimilar split, where entire clusters of drugs and proteins are held out during training. This uniform decline across classical and deep learning methods demonstrates the inherent difficulty of out-of-distribution generalization in DTI affinity prediction, and suggests that the limitations are at least in part data-inherent. The employed datasets do not provide the coverage or diversity required to learn generalizable drug–protein interaction mechanisms. Furthermore, models built on pretrained transformers (DTIAM, LEP-AD variants, Random Forest/XGBoost with ChemBERTa + ESM-3) yield best performance in most similar and dissimilar splits, substantially outperforming CNN-based or cross-attention architectures. This suggests that the large-scale pretraining indeed captures transferable structural signals, enabling better generalization under distribution shift. When considering the size of the training data, distinct trends emerge between small and large datasets. On the small datasets (Davis, Metz, and DTC), transformer-based models—particularly DTIAM and LEP-AD_esm3_concat—achieve the highest number of wins, suggesting that their representation learning mechanisms are well suited to settings with limited training samples. On the large datasets (Stitch, ToxCast, and KIBA), DTIAM continues to dominate, while the LEP-AD variants also remain competitive, with LEP-AD_esm3_concat and LEP-AD_esm3_sum securing multiple wins across metrics and splits. In contrast, traditional machine learning models such as Random Forest Regressor and GTB_DTI achieve fewer wins overall, indicating reduced scalability as dataset size increases. Transformer representations are especially advantageous in low-data or heterogeneous-data regimes, because they reduce the burden on dataset-specific training. To assess robustness to training variability, we performed three independent training replicates on the Davis dataset, confirming that replicate runs do not alter the overall performance trends or benchmark conclusions (Supplementary Fig. 1).

### Experimental literature validation of LEP-AD predictions

We evaluate a series of compounds by comparing their experimental ranks, as reported in the literature, with our predicted ranks to validate our predictive model's accuracy in ranking these compounds based on their binding affinities, measured through IC50 and KD values. We aim to assess the model's reliability by correlating predicted and experimental ranks. Because our experimental validation uses pKi measurements, we train LEP-AD on a combined DTC and Metz dataset to match the measurement type used in our experiments.

We divided the data into quartiles to evaluate the correlation between the predicted ranks (P. Rank) and the experimental ranks (E. Rank) more effectively. We calculate each quartile’s Spearman Rank Correlation Coefficient to assess the monotonic relationships within each category. For the first quartile (Q1), the Spearman Rank Correlation Coefficient (ρ = 0.90) indicates a strong positive monotonic relationship. The corresponding p-value (0.037) shows that this correlation is statistically significant, suggesting that the model’s predictions are reliable within this lowest-ranked group. In the second quartile (Q2), the Spearman rho (ρ = 0.667) suggests a moderate positive monotonic relationship. However, the p-value (0.219) indicates that this correlation is not statistically significant, implying that the model’s predictive reliability weakens in this range. For the third quartile (Q3), the Spearman Rank Correlation Coefficient (ρ = –0.051) indicates no meaningful monotonic relationship between predicted and experimental ranks. The very high p-value (0.934) confirms that this correlation is not statistically significant, showing that the model performs poorly within this category. In the fourth quartile (Q4), which includes the highest predicted rankings, the Spearman rho (ρ = 0.10) reflects a very weak positive monotonic relationship, and the p-value (0.873) shows that this correlation is not statistically significant. Thus, unlike the first quartile, the model offers limited predictive reliability in the highest range of predicted ranks.

Our analysis reveals that in experimental scenarios, as shown in Table 1, the influence of drugs introduces variability in drug–target interactions as determined by our tool. The prediction model performs well for the lowest predicted ranks, where we observe a strong and statistically significant correlation between predicted and experimental rankings. However, for the middle and highest predicted ranks, the correlations are weak and not statistically significant, indicating reduced predictive reliability in these ranges. For example, the compound Src with Dasatinib, ranked first in both predicted and experimental ranks, demonstrated a very low IC50 value of 0.8, indicating a high binding affinity and validating our prediction model's accuracy. Another strong example is Src with UM-164 (ab286966), ranked second in predicted rank and third in experimental rank, with a KD value of 2.7, indicating an accurate prediction. Additional successful cases include Src (Residues 270–523) with UM-164, which shows perfect agreement between predicted and experimental ranks, and Src with WH-4–023 (AB282423), where the predicted and experimental rankings remain closely aligned with the reported IC₅₀ value.

**Table 1:** The target-compound associations, their predicted rankings (P. Rank) based on our model, experimental rankings (E. Rank) from the literature, and corresponding binding values

Target ID	Molecule ID	Predicted Pki	P. rank	E. rank	Reported	M. type	References
Src	Dasatinib	8.7569	**1**	**1**	0.8	IC50	[23]
Src	UM-164 (ab286966)	8.3710	2	3	2.7	KD	[24]
Full Length LYN	Bafetinib	6.6507	7	9	19	IC50	[25]
ShHTL7	Strigol	6.2870	10	14	120	IC50	[26]
ShHTL7	5-Deoxystrigol	6.4550	8	15	120	IC50	[26]
D14	5-Deoxystrigol	5.8191	15	16	330	IC50	[27]
Src (Residues 270–523)	Dasatinib	6.9957	3	2	0.8	IC50	[23]
Src (Residues 270–523)	UM-164 (ab286966)	6.8877	**4**	**4**	2.7	KD	[24]
Src	Src-I1	6.7560	6	10	44	IC50	[28]
Src	SKI-1 (ab120839)	6.7560	6	11	44	IC50	[29]
Src (Residues 270–523)	Src-I1	5.8597	13	12	44	IC50	[28]
Src (Residues 270–523)	SKI-1 (ab120839)	5.8597	**13**	**13**	44	IC50	[29]
Full Length LYN	AZD7762	5.7739	17	8	10	IC50	[30]
D14	GR24	5.8571	14	19	2500	IC50	[27]
ShHTL7	GR24	6.3300	9	18	510	KD	[31]
Src (Residues 270–523)	Saracatinib	6.2556	11	5	2.7	IC50	[32]
Src (Residues 270–523)	WH-4–023 (AB282423)	6.0083	12	6	6	IC50	[33]
Src	WH-4–023 (AB282423)	6.8024	5	7	6	IC50	[33]
ShHTL7	Triton	5.8103	16	17	440	KD	[31]
D14	Triton	5.6812	18	20	No binding	IC50	

Bold values indicate cases where the predicted rank matches the experimental rank

However, the model encounters challenges with other compounds, such as Saracatinib, GR24, and Triton. For Saracatinib, the predicted rank (11) diverges substantially from the experimental rank (5), indicating that the model underestimates its binding potency despite the reported IC₅₀ value of 2.7 μM. GR24 also exhibits large discrepancies across both D14 and ShHTL7 targets, where predicted ranks (14 and 9) differ markedly from experimental rankings (19 and 18), showing that the model struggles to capture the affinity of strigolactone-type compounds. We observe that LEP-AD could not distinguish the experimentally validated interaction between Triton and ShHTL7 from the non-binding of Triton to D14. This result may be explained by ShHTL7 and D14 being structurally homologous proteins. Triton binding to ShHTL7 is of relatively low affinity (the association constant is in the micromolar range) and requires slow structural dynamics [31]. Conversely, high-affinity drugs requiring less structural changes were successfully accurately predicted among the 40 experiments (e.g., Dasatinib-Src). It's important to note that the prediction model while demonstrating high accuracy in some cases, may not fully account for all molecular interactions or the structural dynamics of the compounds. This can lead to inaccuracies, particularly in the middle ranges. The variability in experimental conditions and measurement techniques across different studies can also impact the reported binding affinities, contributing to the observed discrepancies.

### Experimental validation of three ATP-competitive inhibitors

After comparing the predicted ranks with the experimental values reported in the literature, we conducted additional experimental validation on three ATP-competitive Src kinase inhibitors: Dasatinib, UM-164, and Saracatinib. These inhibitors were selected due to their high predicted binding affinities and their significance in previous studies. Focusing on a smaller, well-characterized subset allows for a detailed and rigorous experimental assessment without the extensive resources required for a broader validation. The experimental evaluation involved the following key steps:

- ATP Michaelis–Menten Constant (Km) Determination: establishing the baseline enzyme kinetics of Src kinase ensures that the subsequent IC50 measurements are accurate and reliable.
- IC50 Kinase Inhibitor Assay (ADPGlo) for Src ATP-Competitive Inhibitors: this assay determines the inhibitory potency of the compounds, providing a direct measure of their effectiveness in inhibiting Src kinase activity.
- Logarithmic Transformation of the IC50 Kinase Inhibitor Assay (ADPGlo): transforming the IC50 data logarithmically allows for a more precise comparison of the inhibitors’ potencies across a wide range of values.
- Comparison of IC50 and pKi Values: this comparison facilitates the validation of the computational predictions pKi against the experimental IC50 values, as this highlights the accuracy of the prediction model.
- Three-Point Correlation of the Src Inhibitor pKi Predicted vs. Experiment: this analysis directly correlates predicted and experimental values, demonstrating the model’s reliability.

**Fig. 5:**
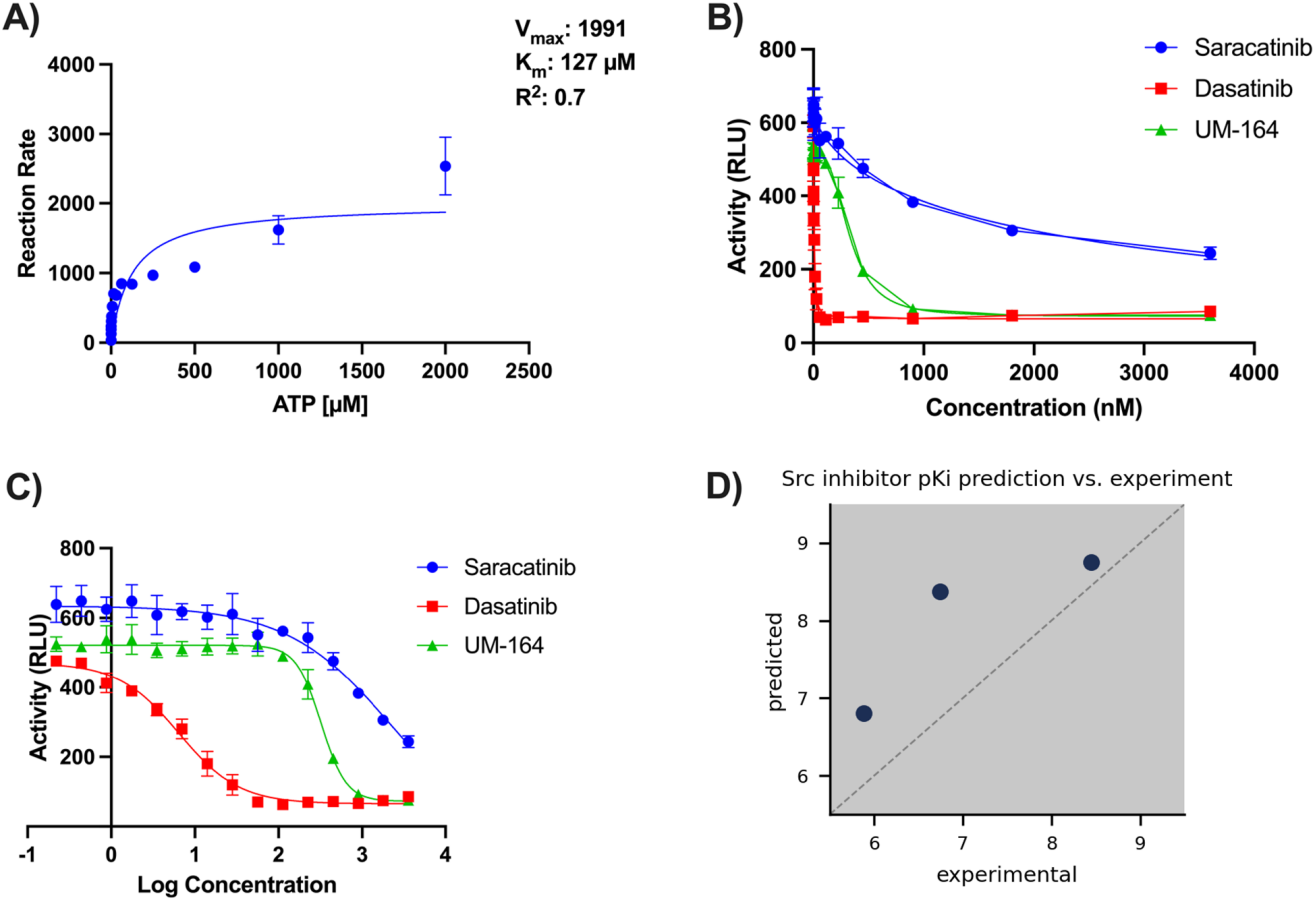
Experimental evaluation of three ATP-Competitive Src Kinase inhibitors. **A**) Km determination, **B**) ADPGlo for Src ATP-Competitive Inhibitors (linear scale), **C**) ADPGlo for Src ATP-Competitive Inhibitors (log scale), and **D**) Three-point correlation of the Src inhibitor pKi predicted vs. experiment. The Src Kinase and ATP concentrations used in the assay are 50 nM and 100 µM, respectively.

Because our experimental validation relies on pKi-based measurements, we ensured that the model was trained on data using the same measurement scale by combining the DTC and Metz datasets, both of which report affinity in pKi. The result is shown in Fig. 5 and Table 2. Table 2 compares the experimental and predicted IC₅₀ and pKi values, along with the relative rankings, of three ATP-competitive Src kinase inhibitors: Dasatinib, UM-164, and Saracatinib. Dasatinib is ranked first in both our predictions and experimental evaluations, UM-164 is ranked second, and Saracatinib is ranked third. While the experimental IC₅₀ and pKi values do not match the predicted values exactly, they follow the same overall potency order reflected in our model’s predictions. Additionally, we compare the performance of the benchmark methods on the experimental data, and the results are shown in Supplementary Table 1.

**Table 2:** Experimental evaluation of three ATP-Competitive Src Kinase inhibitors

Rank	Inhibitor	Experimental		Predicted	
		IC50 (M)	pKi (M)	IC50 (M)	pKi (M)
1	Dasatinib	6.40E-09	8.446	3.12E-09	8.757
2	UM-164	3.23E-07	6.743	7.60E-09	8.371
3	Saracatinib	2.33E-06	5.884	2.78E-07	6.808

## Model interpretation

**Fig. 6:**
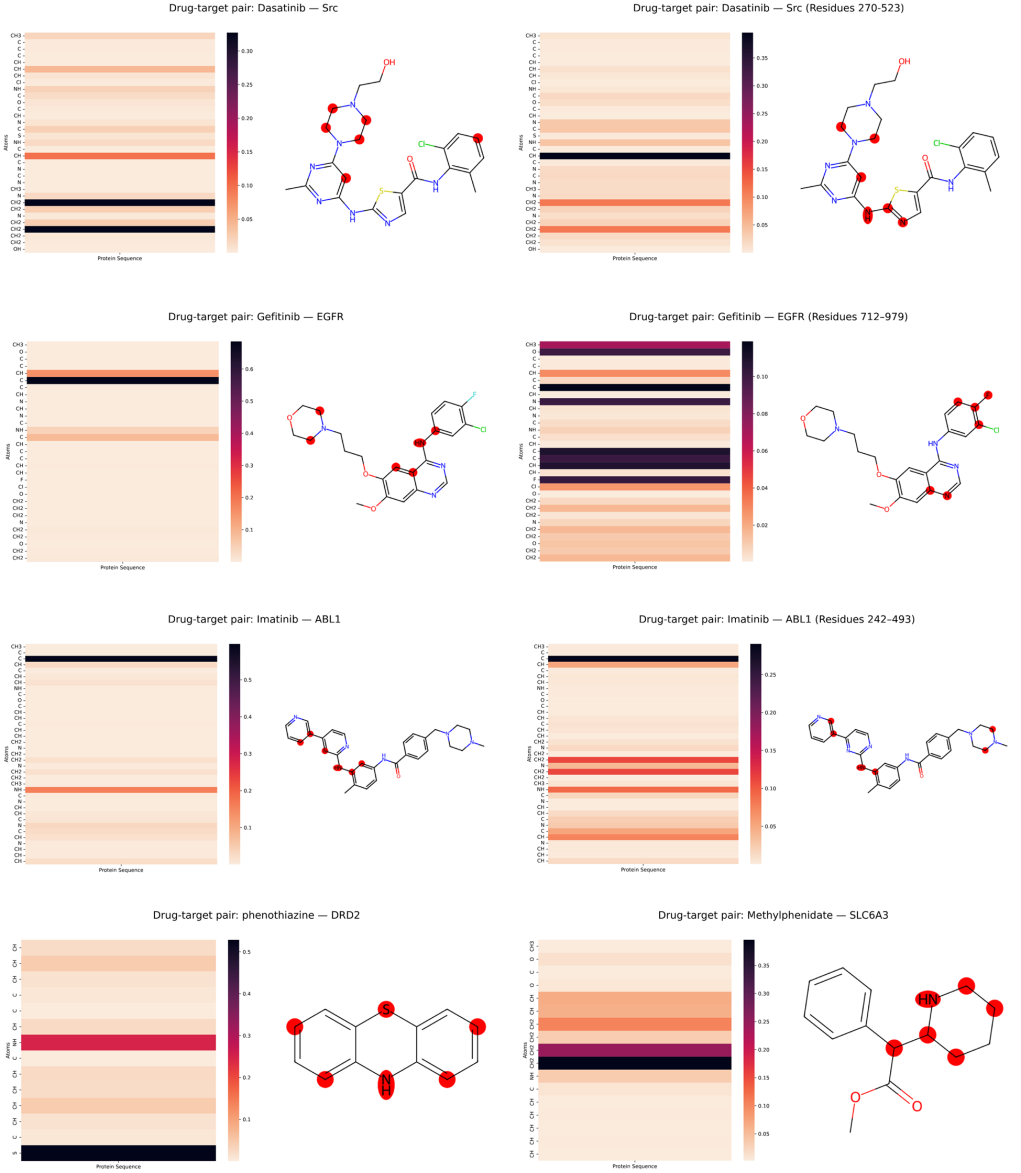
Cross-attention analysis between ligand atoms and protein representations for selected drug–target pairs. Heatmaps show cross-attention weights between atom-level drug embeddings from the GAT encoder and a pooled protein representation derived from ESM-3, yielding one attention score per ligand atom. The left panels display the attention weights, while the right panels highlight the corresponding high-attention atoms on the molecular structures

To gain qualitative insight into model behavior, we visualized cross-attention weights between ligand atoms and protein sequence representations for selected drug–target pairs (Fig. 6). On the ligand side, the highest attention weights consistently correspond to chemically meaningful substructures, such as hinge-binding heterocycles, aromatic rings, and amine-containing moieties, which are well known to drive target engagement.

On the protein side, attention weights tend to concentrate over broader sequence regions corresponding to domains known to be involved in ligand binding, rather than being uniformly distributed across the sequence. For example, in the gefitinib–EGFR case, higher attention is associated with the kinase catalytic domain encompassing the hinge region, while ligand-side attention highlights the quinazoline core and adjacent anilide group known to engage the ATP-binding cleft (PMID: 37111291). Similarly, imatinib and dasatinib show ligand-side emphasis on heterocyclic hinge-binding rings and amide linkers, with protein-side attention enriched over the Abl/Src kinase domain, consistent with known differences in conformational preference (PMID: 23226582).

For transporter and GPCR examples, such as methylphenidate, SLC6A3 and phenothiazine, DRD2, ligand-side attention emphasizes aromatic and amine moieties commonly involved in neurotransmitter recognition, while protein-side attention is enriched over transmembrane regions previously implicated in ligand binding (PMID: 18568020; PMID: 35889317). These patterns indicate that the model attends to biologically plausible features at a coarse, domain-level resolution.

Comparing full-length and truncated protein sequences, we observe that truncation increases the contrast of attention patterns within the catalytic or functional domain, whereas full-length sequences exhibit lower peak intensities and additional secondary bands. This effect arises from the normalization of attention weights via the softmax operation across a larger number of sequence tokens, such that longer sequences distribute attention more broadly. Importantly, this does not imply improved spatial localization but rather reflects how sequence length influences the relative weighting of informative regions.

Overall, these visualizations should be interpreted as heuristic indicators of model focus, highlighting consistency with known binding-related regions and ligand pharmacophores. Attention weights capture statistical correlations within the learned representations and do not imply residue-level localization or physical interaction mechanisms; they complement, but cannot replace, structure-based or experimental analyses.

## Discussion

In this study we introduced LEP‑AD and evaluated its performance under similarity‑aware data splits that enforce a meaningful separation between training and test drugs and proteins. Across multiple benchmarks, all methods, including LEP‑AD and contemporary deep learning baselines, exhibited marked drops in performance when evaluated on dissimilar drugs and proteins. This suggests that out‑of‑distribution generalisation remains a major unsolved challenge in drug–target interaction modelling. In small datasets such as Davis, the limited diversity of available examples amplifies this effect and can even make simpler baseline models appear competitive.

Our experiments further indicate that the quality of pretrained protein and ligand embeddings is the primary driver of predictive performance, particularly in low‑data regimes. By contrast, the choice of fusion strategy has a more limited impact: simple concatenation tends to yield slightly better predictive performance, while cross‑attention offers interpretability but does not consistently improve accuracy under distribution shifts. This finding, along with the overall difficulty of generalising to dissimilar drugs and proteins, raises doubts that ever more elaborate architectures alone will solve the DTI prediction problem without addressing underlying data limitations.

Cross‑attention and related attention mechanisms are widely used as tools for exploring model focus. In LEP‑AD, visualisations of cross‑attention weights highlight ligand atoms corresponding to key pharmacophores and align with known functional regions of the protein, such as kinase hinge regions and transmembrane segments. Such qualitative correspondence suggests that the model attends to chemically meaningful features. However, it is important to interpret these patterns cautiously. Attention weights indicate statistical correlations in the model’s learned representations; they do not quantify binding energies or establish causality. Studies of related models show that only a small fraction of high‑attention residues correspond to experimentally validated binding sites, and one‑dimensional sequence inputs can obscure three‑dimensional context. Thus, attention‑based interpretability provides heuristic insights rather than residue‑level localisation and should be complemented by structural analysis or mutagenesis experiments.

Finally, by benchmarking across diverse splits and highlighting the limitations of current methods, our work underscores the need for richer and more representative datasets and for approaches that combine strong pretrained representations with explicit interaction modelling. Out‑of‑distribution prediction, data bias, and the lack of reliable interpretability remain open challenges for DTI modelling that warrant further investigation.

## Methods

### LEP-AD overview

The proposed LEP-AD architecture is designed to predict protein–ligand binding affinity by integrating drug structure and protein sequence representations, as illustrated in Fig. 1. The drug representation is obtained by first transforming the molecular structure into a graph, where atoms are represented as nodes ($V$) and chemical bonds as edges ($E$). To capture the topological and chemical context of the molecule, either a graph attention–based neural network (GATv2) [16] or a graph transformer operator [15] is applied. These models enable the computation of attention-weighted interactions between neighboring nodes, resulting in expressive molecular representations that encode the structural properties of the molecule. The choice between the two methods is determined through hyperparameter optimization for each dataset.

Graph transformer and GAT are categorized as message-passing GNNs, in which the feature representation of a given node ${h}_{i}$ is updated based on its neighbors in ${\mathcal{N}}_{i}=\{j\in V\mid (j,i)\in E\}$:

$${{h}{\prime}}_{i}= {f}_{\theta }({h}_{i}, \mathrm{AGGREGATE}\left(\{{h}_{j} \right| j\in {\mathcal{N}}_{i}\}))$$

The main distinction between different forms of GNNs lies in the specific design of $f$ and $\mathrm{AGGREGATE}$ functions. For the GAT-based methods employed in this study, the following design is used:

$${{h}^{{{\prime}}}}_{i}= {}_{c=1}{}^{C}||\sum_{j \in {\mathcal{N}}_{i}}\sigma \left({\alpha }_{i,j}^{c}{{\boldsymbol{W}}}^{c}{h}_{j}\right),$$

Where $\parallel$ denotes concatenation, ${\alpha }_{i,j}^{c}$ represents the attention score between nodes $i$ and $j$ for head $c$, ${{\boldsymbol{W}}}^{c}$ is a learnable weight matrix for head $c$, and $\sigma$ is an activation function. This formulation provides a general representation of the multi-head attention mechanism on graph structures [34], where each node attends to its neighbors as a query node. GAT-based algorithms are distinguished by the attention function used to compute ${\alpha }_{i,j}^{c}$. In GATv2, the attention coefficient is computed through an additive attention function as:

$${e}_{i,j}^{c}= {{\boldsymbol{a}}}^{\rm T}LeakyReLU({{\boldsymbol{W}}}^{c}\cdot [{h}_{i}||{h}_{j}])$$

$${\alpha }_{i,j}^{c}= \frac{exp({e}_{i,j}^{c})}{\sum_{l\in {\mathcal{N}}_{i}}exp({e}_{i,l}^{c})}$$

while graph transformer uses scaled dot-product attention [15] to compute the unnormalized attention score:

$${e}_{i,j}^{c}= \frac{({{\boldsymbol{W}}}_{Q}^{c}{h}_{i}){({{\boldsymbol{W}}}_{K}^{c}{h}_{j})}^{T}}{\sqrt{{d}_{c}}}$$

Using these learnable scores, each method learns an embedding matrix that encodes the structural context of each node in the graph. Since the task involves predicting binding affinity at the graph level and integrating the molecular representation with the protein representation, a graph pooling operation is applied to transform the node embeddings into a single fixed-size molecular embedding for downstream prediction:

$${h}_{G}= \mathit{POOL}(\{{h}_{i}| i\in V\}),$$

where *POOL* denotes an aggregation function that can be the average, sum, or maximum over each feature dimension across the nodes. Similar to the GAT modules, the choice of pooling function is determined through hyperparameter optimization for each dataset. In addition to these pooling operations, our framework also supports a no-pooling option, in which the full set of node-level embeddings is retained and passed directly into the cross-attention fusion module. This allows the model to exploit the full structural resolution of the molecule. The final drug representation module consists of three GAT layers, each followed by a ReLU activation and a pooling layer (or no pooling when the full graph representation is selected). The resulting graph-level representation is subsequently integrated with the protein representation to enable binding affinity prediction.

For the protein representation, models from the ESM-3 family [7] are employed. ESM-3 is a state-of-the-art protein language model trained on large-scale protein sequence databases using self-supervised learning objectives. It is designed to model the three core properties of proteins—sequence, structure, and function—within a unified framework based on Transformer architecture. In this model, sequence tokens encode the amino acid composition, structural tokens discretize three-dimensional atomic coordinates into a vocabulary of spatial states, and functional tokens represent higher-level biological information associated with the protein. In contrast, ESMC relies solely on sequence tokens and serves as a computationally efficient alternative to ESM-3, replacing the earlier ESM-2 model while maintaining high-quality sequence representations. Since binding affinity is strongly influenced by the three-dimensional conformation and functional context of proteins, ESM-3 is hypothesized to provide more informative representations than ESMC, potentially leading to improved predictive performance. To test this hypothesis, we compared the performance obtained using ESM-3 and ESMC embeddings to predict binding affinity values across six drug–target pair datasets (Supplementary Fig. 2). Across multiple hyperparameter configurations, we observe that overall performance remains largely consistent across protein representation choices, with ESM-3 providing a slight to modest advantage over ESMC. Based on this observation, we adopt ESM-3 in the main experiments, as it is pretrained on a broader range of information extending beyond primary sequence to include structural and functional context. Accordingly, ESM-3 is used to derive the final amino acid sequence representations in our model. Since the protein embedding matrix is high dimensional, a single-layer neural network is applied to project it into the same hidden dimension as the drug representations.

To integrate the molecular and protein representations, a fusion layer combines the two embeddings into a unified representation for binding affinity prediction. Depending on the configuration, the fusion is performed using one of several strategies: simple concatenation of the two vectors, element-wise summation, or a multi-head cross-attention mechanism that allows the model to dynamically weight and align features across modalities. Concatenation and summation provide efficient and low-complexity fusion, while cross-attention offers greater flexibility to model complex interactions between the molecular structure and the protein sequence:

$$F\left(P, D\right)=Attn\left(P,D\right) || Attn(D,P),$$

$$Attn\left(Q, K, V\right)=softmax\left(\frac{\left(Q{W}_{Q}\right){\left(K{W}_{K}\right)}^{T}}{\sqrt{{d}_{K}}}\right)V.$$

Here, $P$ represents the protein embedding and $D$ represents the drug (molecule) embedding. $Attn$ denotes the cross-attention operation, which is applied bidirectionally to capture mutual dependencies between the two modalities. In $Attn(P,D)$, the protein embedding serves as the query ($Q$), while the drug embedding provides the keys ($K$) and values ($V$), allowing the model to learn how protein features are influenced by molecular structure. Conversely, in $Attn(D,P)$, the drug embedding serves as the query and the protein embedding as the keys and values, enabling the model to encode how molecular features depend on protein context. Concatenating these two attention outputs results in a unified interaction representation that captures complementary protein–ligand relationships. Finally, a three-layer neural network is applied to predict the binding affinity value.

### Datasets

We utilized several datasets for our evaluation and comparison, as summarized in Supplementary Table 2. These datasets include Davis, KIBA, Drug Target Commons (DTC), Metz, ToxCast, and STITCH. The Davis dataset is renowned for its comprehensive coverage of kinase inhibitors and their dissociation constants (pKd), making it crucial for evaluating kinase-target interactions. The KIBA dataset integrates multiple bioactivity measures, providing a robust benchmark for drug-target interaction predictions. The DTC (pKi) and Metz (pKi) datasets are pivotal for evaluating kinase inhibitor interactions, offering insights into the binding affinities of inhibitors. The STITCH dataset offers extensive interaction data between chemicals and proteins, facilitating studying chemical-protein interactions. The ToxCast dataset provides high-throughput screening data essential for assessing the toxicological profiles of various compounds.

### Similarity-based cross-validation

Conventional random k-fold cross-validation splits often lead to data leakage because compounds in the test fold may be highly similar to those in the training folds. This enables machine learning models to achieve apparently strong performance by exploiting global similarity rather than learning meaningful, generalizable patterns. As a result, the reported metrics are often artificially inflated, providing a misleading estimate of a model’s true predictive power on unseen compounds or targets.

To more rigorously assess the generalization capability of our model and the benchmark methods, we employ a clustering-based cross-validation strategy that partitions the dataset according to protein similarity and drug similarity. This evaluation requires embedding representations for both drugs and proteins, with each representation preserving the structural and functional similarities intrinsic to its respective modality. Specifically, the drug representations are obtained from the pretrained ChemBERTa model, which is based on the RoBERTa architecture and trained on large-scale chemical corpora to capture molecular structure and context [8]. Protein representations are derived from the pretrained ESM3 model, which learns informative embeddings from amino acid sequences, effectively encoding protein structure and function [7]. The ChemBERTa embeddings have a dimensionality of 768, while the ESM3 embeddings have a dimensionality of 1,536. We standardize the embeddings of both modalities using z-score normalization before applying the Louvain clustering algorithm. The Louvain algorithm is particularly well suited for this task because it is both highly efficient and scalable, making it practical for datasets containing a large number of drugs and proteins. Moreover, its modularity-based optimization enables the identification of meaningful community structures without requiring the number of clusters to be specified in advance, which is especially advantageous when dealing with complex biological datasets. For both modalities and across all six datasets, we use either 1.0 or 1.5 as the resolution parameter values. This parameter controls the granularity of the clustering process, where lower values lead to fewer, larger clusters and higher values yield more fine-grained cluster partitions. In our analysis, we aim to generate a sufficiently large number of clusters to enable the removal of entire clusters from the training set, thereby simulating out-of-distribution scenarios. At the same time, each cluster must contain an adequate number of samples to allow the selection of representative test examples, enabling us to also simulate in-distribution prediction settings. To operationalize this, we construct two data splits: **“similar_split”**, in which drugs and proteins are sampled from each cluster, and **“dissimilar_split”**, in which entire clusters of drugs and proteins are held out. The UMAP panels in Supplementary Figs. 3 and 4 illustrate the distribution and clustering structure of the drug and protein embeddings used to construct our similarity-based cross-validation splits across all six datasets. For each dataset, the first UMAP projection shows the Louvain clusters obtained from the ChemBERTa drug embeddings, while the middle and right projections display the dissimilar and similar splits for the drugs (Supplementary Fig. 3), and similarly for the ESM-3 protein embeddings (Supplementary Fig. 4).

### Hyper-parameters

To optimize model performance across different datasets, we employed automated hyperparameter optimization using Optuna [35]. A separate hyperparameter search was conducted for each dataset to account for variations in data scale and distribution. The search space encompassed architectural, training, and regularization parameters.

Specifically, the following hyperparameters were tuned:

- **Batch size**: $$\{128, 256, 512\}$$
- **Hidden dimension (GNN)**: $\{128, 256, 512\}$
- **Hidden dimension (MLP)**: $\{128, 256, 512, 1024\}$
- **Number of attention heads (fusion layer)**: $\{1, 2, 4\}$
- **Number of attention heads (GNN)**: $\{4, 8\}$
- **GNN layer type**: {GATv2, TransformerConv}
- **Dropout rate**: $$\{0.0, 0.1, 0.2\}$$
- **Learning rate**: $$\{5\times {10}^{-4},{ 10}^{-4}, 5\times {10}^{-3},{ 10}^{-3}\}$$

The optimal hyperparameter configurations identified for each dataset are reported in Supplementary Table 3.

### Evaluation metrics

The **Mean Squared Error (MSE)** is a widely used metric to quantify the difference between predicted and actual values. For a dataset with $n$ samples, the MSE is defined as the average of the squared differences between the predicted values ${p}_{i}$ and the corresponding true values ${y}_{i}$:

$${\mathrm{MSE}}=\frac{1}{n}\sum_{i=1}^{n}({p}_{i}-{y}_{i}{)}^{2}$$

A smaller MSE indicates that the model’s predictions are closer to the true observations, reflecting better predictive accuracy. To further assess model performance, we use the ${r}_{m}^{2}$ metric [36], defined as:

$${r}_{m}^{2}={r}^{2}(1-\sqrt{\mid {r}^{2}-{r}_{0}^{2}\mid })$$

where ${r}^{2}$ and ${r}_{0}^{2}$ represent the squared correlation coefficients between the observed and predicted values—with and without intercept, respectively. In Quantitative Structure–Activity Relationship (**QSAR**) modeling, predictions are typically considered acceptable when ${r}^{2}\ge 0.5$.

Additionally, the **Concordance Index (CI)** measures the model’s ability to correctly rank predicted values relative to their true values. It quantifies the probability that, for any pair of samples, the sample with the higher true value also has a higher predicted value. The CI is computed as:

$${\mathrm{CI}}=\frac{1}{Z}\sum_{x>y}h({b}_{x}-{b}_{y})$$

where $Z$ is the total number of comparable pairs, ${b}_{x}$ and ${b}_{y}$ are predicted values, and $h(\cdot )$ is the Heaviside step function. A higher CI indicates better ranking consistency between predicted and true values.

### Deep learning models included in the benchmark

- **DTIAM**: The protein sequence is encoded using a transformer model pretrained in a self-supervised manner to learn contextualized residue representations via masked language modeling and contact prediction. In parallel, the drug is represented as a molecular graph that is decomposed into chemically meaningful substructures, which are encoded using a transformer pretrained with masked substructure prediction, molecular descriptor prediction, and functional property prediction objectives. The resulting protein and drug representations are integrated through feature concatenation and used as input to downstream prediction models, including multilayer perceptrons and ensemble-based learners, for drug–target interaction and binding affinity prediction.
- **TDGraphDTA**: The protein sequence is encoded using a multi-scale convolutional neural network to capture contextual residue patterns, while the drug molecule is represented as a graph and processed through a graph diffusion mechanism to learn structural features. The resulting protein sequence embeddings and drug graph embeddings are integrated using a multi-head cross-attention module, in which both modalities are linearly projected into query, key, and value representations. Two parallel cross-attention layers model bidirectional interactions between the drug and protein features, and their outputs are concatenated and passed to a multilayer perceptron to produce the final binding affinity prediction.
- **GTB_DTI**: The protein sequence is encoded using a multi-scale convolutional neural network to capture contextual residue features. The drug is represented through two complementary modalities: a molecular graph and a SMILES sequence. The molecular graph is processed using a graph-based encoder with stacked attention layers and global pooling to learn structure-aware representations, while the SMILES sequence is encoded via a convolutional neural network followed by max pooling. The resulting graph-based and sequence-based drug representations are combined through multilayer perceptrons, producing a unified drug embedding. Finally, the drug and protein embeddings are concatenated and passed to task-specific prediction heads for drug–target interaction and binding affinity prediction.
- **CSDTI**: A cross-attention–based interaction framework is employed to fuse deep representations of drugs and proteins. The drug molecule is represented as a molecular graph and encoded using a graph-based drug molecule aggregator to capture higher-order structural dependencies, while the protein sequence is encoded using a one-dimensional convolutional neural network to learn contextual residue features. In the interaction module, the drug representation is projected as the query, and different projections of the protein representation serve as the key and value in a cross-attention block, enabling fine-grained modeling of drug–protein interactions. The resulting interaction-aware representations are then passed to a prediction head to estimate drug–target interaction probabilities.
- **IMAEN**: A molecular structure augmentation mechanism is employed to enrich drug representations by fully aggregating neighborhood information from the molecular graph. The augmented drug graph is processed using a multi-scale graph convolutional network, followed by a self-attention module to capture long-range dependencies among molecular features. In parallel, the protein sequence is encoded using a multi-scale convolutional neural network to extract residue-level patterns, with an additional self-attention layer to model contextual relationships along the sequence. The resulting drug and protein embeddings are concatenated and passed to a multilayer perceptron to produce the final drug–target interaction prediction.

### Experimental validation of predicted pKi

Using the ADP-Glo phosphorylation assay (Promega), we determined the Michalis-Menten constant (*K*_*m*_) for the substrate (ATP) with a peptide substrate phosphorylated by Src kinase. The 50 nM Src Kinase and one µM peptide substrate concentrations were constant, while ATP concentration was variable. The calculated ATP *K*_*m*_ was 127 ± 133 μM, close to the reported ATP *K*_*m*_ of 130 µM in the literature [37]. Next, we conducted an IC50 assay of three ATP-competitive Src kinase inhibitors: Saracatinib, Dasatinib, and UM-164. The Src Kinase and ATP concentrations used in the assay are 50 nM and 100 µM, respectively. The inhibitor concentrations were from 0.22 to 3600 nM through serial dilutions. The reaction buffer consisted of 40 mM Tris–HCl (pH 7.5), 20 mM MgCl2, 2 mM MnCl2, BSA 0.1 mg/mL, and 2 mM DTT. We followed the ADP-Glo assay protocol to determine the IC50. First, we incubated the reaction of 5 µL of Src kinase, peptide substrate, ATP, and the inhibitor for 3 h at room temperature. We added 5 μL of ADP-Glo reagent to deplete the remaining ATP and incubated for 40 min at room temperature. Then, we added 10 μL of kinase detection reagent and incubated for 30 min. After that, a bioluminescent measurement was taken using the Tecan M1000 plate reader. The IC50 was determined from a non-linear regression analysis of the “[Inhibitor] vs. response” using Prism/GraphPad version 10.0. The *K*_*i*_ was calculated using the Cheng-Prusoff equation: Ki = IC50/(1 + ([S])/Km) and pKi = − log(Ki). The Cheng-Prusoff equation [38] is applicable when the inhibitor is competitive. Hence, in this case, the three inhibitors are ATP-competitive, where the ATP is considered the substrate [S].

## Supplementary Information

Additional file 1.

## Data Availability

ESM embeddings, along with all datasets and code used in this study, are available for download at: https://github.com/reem12345/LEP-AD.
