# Supplementary material for: LEP-AD: language embedding of proteins and attention to drugs predicts drug-target interactions
Source: J Cheminform. 2026 Apr 27;18:101. doi: 10.1186/s13321-026-01167-9 (PMC13397736; doi:10.1186/s13321-026-01167-9)
Supplement: Supplementary file 1 — Additional file 1. [file 13321_2026_1167_MOESM1_ESM.docx]

# **Supplementary Figures:**


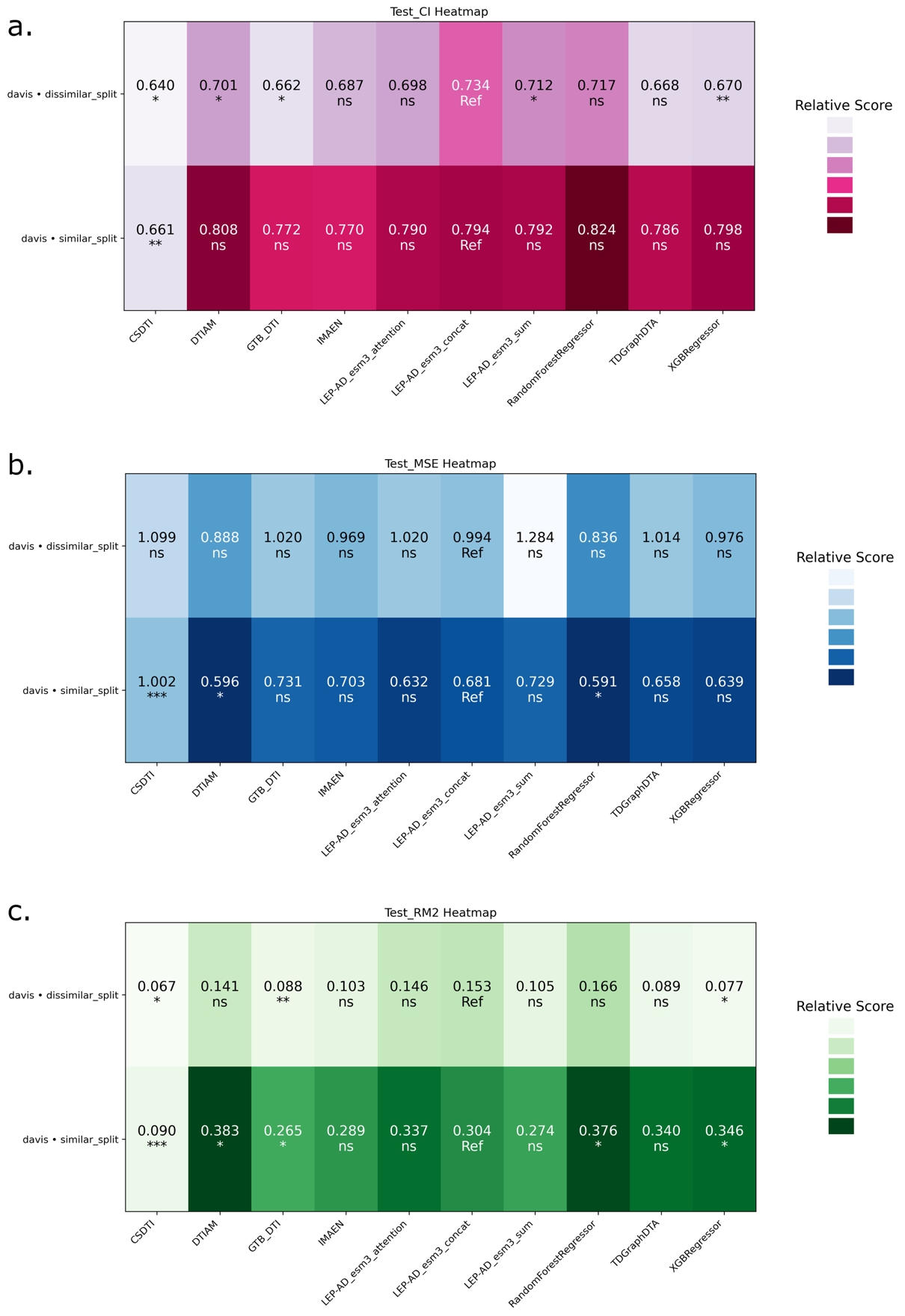


**Supplementary Fig. 1 | Benchmark performance of predictive models on the Davis dataset across similar and dissimilar splits.** **(a)** Concordance Index (CI), **(b)** Mean Squared Error (MSE), and **(c)** $r_{m}^{2}$ heatmaps for ten predictive models evaluated on the Davis dataset under similar and dissimilar splits. Each cell shows the model’s mean performance across three independent training replicates, with darker colors indicating better relative performance (per-dataset normalization). Statistical significance is computed using paired t-tests comparing each model against the reference method LEP-AD_esm3_concat. Significance levels are shown beneath each value (ns: not significant; * p < 0.05; ** p < 0.01; *** p < 0.001), and “Ref” marks the reference model.

**Supplementary Fig. 2|** **Comparison of LEP-AD performance using ESMC-600M versus ESM-3 protein embeddings under the same baseline fusion strategy.** Panels **a**, **b**, and **c** display heatmaps of the **concordance index (CI)**, **RM2**, and **mean squared error (MSE),** respectively, for LEP-AD when using two different protein embedding models—**ESMC-600M** and **ESM-3**—while keeping the **same baseline fusion strategy** fixed. Results are shown for all datasets (DTC, Metz, Stitch, ToxCast, Davis, and KIBA) under both **similar** and **dissimilar** cross-validation splits. Colors are normalized **per dataset** to highlight performance differences between the two embedding types within each dataset. Original metric values appear inside each cell, and the color bars indicate the relative score range for each metric.

**Supplementary Fig. 3 | UMAP visualization of ChemBERTa drug embeddings across six datasets under similar and dissimilar split strategies.** UMAP projections are shown for: (a) ToxCast, (b) Stitch, (c) DTC, (d) KIBA, (e) Metz, and (f) Davis. For each dataset, three panels are provided: the left panel displays Louvain clusters computed from the ChemBERTa embeddings; the middle panel illustrates the train/test assignment for the dissimilar split, where test-set compounds correspond to entire outlier clusters; and the right panel depicts the train/test assignment under the similar split, where test compounds are sampled from each cluster in proportion to its size.

**Supplementary Fig. 4 | UMAP visualization of ESM-3 protein embeddings across six datasets under similar and dissimilar split strategies.** UMAP projections are shown for: (a) ToxCast, (b) Stitch, (c) DTC, (d) KIBA, (e) Metz, and (f) Davis. For each dataset, three panels are provided: the left panel displays Louvain clusters computed from the ESM-3 embeddings; the middle panel illustrates the train/test assignment for the dissimilar split, where test-set proteins correspond to entire outlier clusters; and the right panel depicts the train/test assignment under the similar split, where test proteins are sampled from each cluster in proportion to its size.

# **Supplementary Tables:**

**Supplementary Table 1:** **Experimental validation benchmarking on kinase inhibitors.** This table reports the predicted pKi values for three experimentally tested compounds—Dasatinib, UM-164, and Saracatinib—using LEP-AD and five baseline models (CSDTI, DTIAM, IMAEN, TDGraphDTA).

| Model | Dasatinib | UM-164 | Saracatinib | |
| --- | --- | --- | --- | --- |
| Experimental | 8.446 | 6.743 | 5.884 |  |
| CSDTI | 6.140 | 6.554 | 5.817 |  |
| DTIAM | 8.048 | 6.622 | 6.398 |  |
| GTB_DTI | 8.332 | 7.111 | 6.500 |  |
| IMAEN | 8.808 | 7.664 | 7.019 |  |
| LEP-AD | 8.757 | 8.371 | 6.808 |  |
| TDGraphDTA | 9.254 | 7.849 | 6.809 |  |

**Supplementary Table 2:** Dataset statistics.

| Dataset | Number of Drugs | Number of Targets | Total number of drug-target pairs | |
| --- | --- | --- | --- | --- |
| Davis | 68 | 442 | 30056 |  |
| KIBA | 2111 | 229 | 118254 |  |
| DTC | 5983 | 118 | 67894 |  |
| Metz | 1471 | 170 | 35307 |  |
| ToxCast | 7657 | 328 | 342869 |  |
| STITCH | 724471 | 15258 | 1244420 |  |

**Supplementary Table 3:** Optimal hyperparameter configurations for each dataset after hyperparameter optimization.

| Dataset | Batch Size | Hidden Dim (GNN) | Hidden Dim (MLP) | Fusion Heads | GNN Heads | GNN Layer | Dropout | Learning Rate |
| --- | --- | --- | --- | --- | --- | --- | --- | --- |
| Davis | 128 | 256 | 128 | 2 | 4 | TransformerConv | 0.2 | 5 × 10⁻⁴ |
| DTC | 256 | 128 | 1024 | 1 | 8 | GATv2 | 0.1 | 5 × 10⁻⁴ |
| KIBA | 128 | 128 | 1024 | 4 | 8 | TransformerConv | 0.1 | 5 × 10⁻⁴ |
| Metz | 256 | 128 | 512 | 1 | 4 | TransformerConv | 0.2 | 5 × 10⁻⁴ |
| ToxCast | 512 | 512 | 1024 | 2 | 8 | TransformerConv | 0.2 | 1 × 10⁻⁴ |
| STITCH | 512 | 512 | 1024 | 2 | 8 | TransformerConv | 0.2 | 1 × 10⁻⁴ |
